# Interrogating the intentions for Aboriginal and Torres Strait Islander health: a narrative review of research outputs since the introduction of Closing the Gap

**DOI:** 10.5694/mja2.51601

**Published:** 2022-06-10

**Authors:** Michelle Kennedy, Jessica Bennett, Sian Maidment, Catherine Chamberlain, Kate Booth, Romany McGuffog, Bree Hobden, Lisa J Whop, Jamie Bryant

**Affiliations:** ^1^ University of Newcastle Newcastle NSW; ^2^ Hunter Medical Research Institute University of Newcastle Newcastle NSW; ^3^ Centre for Health Equity University of Melbourne Melbourne VIC; ^4^ Judith Lumley Centre La Trobe University Melbourne VIC; ^5^ National Centre for Epidemiology and Population Health Australian National University Canberra ACT

**Keywords:** Review article, Publishing, Data collection, Research design

## Abstract

- Despite the “best of intentions”, Australia has fallen short of federal targets to close the gap in disproportionate health outcomes between Aboriginal and non‐Aboriginal Australians.
- We examined 2150 original research articles published over the 12‐year period (from 2008 to 2020), of which 58% used descriptive designs and only 2.6% were randomised controlled trials. There were few national studies. Studies were most commonly conducted in remote settings (28.8%) and focused on specific burdens of disease prevalent in remote areas, such as infectious disease, hearing and vision. Analytic observational designs were used more frequently when addressing burdens of disease, such as cancer and kidney and urinary, respiratory and endocrine diseases.
- The largest number of publications focused on mental and substance use disorders (*n* = 322, 20.5%); infectious diseases (*n* = 222, 14.1%); health services planning, delivery and improvement (*n* = 193, 33.5%); and health and wellbeing (*n* = 170, 29.5%).
- This review is timely given new investments in Aboriginal health, which highlights the importance of Aboriginal researchers, community leadership and research priority. We anticipate future outputs for Aboriginal health research to change significantly from this review, and join calls for a broadening of our intellectual investment in Aboriginal health.

It is not credible to suggest that one of the wealthiest nations in the world cannot solve a health crisis affecting less than 3% of its citizens.^1^

Since the 2005 Social Justice report criticised the Australian Government for not addressing the inadequate life expectancy of Aboriginal and Torres Strait Islander people, national efforts have been made towards closing the gap between Aboriginal and Torres Strait Islander people and non‐Aboriginal Australians. The then Social Justice Commissioner, Professor Tom Calma AO, made three recommendations to address equality in life expectancy: i) a government commitment to achieving equality in health status in 25 years, ii) equality in access to primary care and health infrastructure, and iii) bipartisan support for this commitment.^2^ Improvements in age‐standardised mortality rates of about 10% have been observed for Aboriginal and Torres Strait Islander people since 2006, but similar improvements have occurred for non‐Aboriginal Australians.^3^ Therefore, Australia has fallen short of the federal government’s targets to close the gap in disproportionate health outcomes and life expectancy for Aboriginal and Torres Strait Islander people.^3^

In the Prime Minister’s 2020 Closing the Gap statement to Parliament, he reported “despite the best of intentions; investments in new programs; and bi‐partisan goodwill, Closing the Gap has never really been a partnership with Indigenous people”.^4^ The “best of intentions” for Closing the Gap has been widely questioned in academic literature^5^, ^6^ and mainstream media,^7^, ^8^ including highlighting the lack of Aboriginal and Torres Strait Islander peoples involvement in decision‐making processes^9^ and acknowledgement of Aboriginal Community Controlled Health Services as exemplars of best practice in providing holistic health care to Aboriginal and Torres Strait Islander people.^10^ Over the past 12 years, the reported “investments in new programs” funding has fluctuated,^11^, ^12^ highlighting resources as finite. The Closing the Gap framework identifies the need for research and evaluation to inform effective policies,^13^ with calls for increased research in urban settings^14^ and to evaluate evidence‐based practices, policy and programs.^6^ Early research also explored the extent to which the policy changes informed new models for research conduct.^15^ In 2021, with a reformed agenda for Closing the Gap now established with Aboriginal and Torres Strait Islander people represented by their community‐controlled peak organisations, the Coalition of Peaks^16^ — an Aboriginal‐led research team — felt it timely to interrogate the intentions for Aboriginal and Torres Strait Islander health through a critical review of research outputs since Closing the Gap was established in 2008.

Providing an overview of Aboriginal and Torres Strait Islander health research since 2008 allows for an examination of the scope and characteristics of the knowledge base to inform evidence‐based practices, policies and programs. To date, no review of Indigenous health research outputs have described the scope and characteristics of the research, most notably the burden of disease focus or the research designs being implemented. Previous reviews have identified continual growth in research outputs in the literature,^17^, ^18^ a lack of intervention research,^17^ and a lack of research in urban settings.^19^ The extent of research on a targeted burden of disease, examining research designs used and geographical setting, is crucial knowledge to inform the potential impact of research efforts on informing evidence‐based practices, policies and programs.

Aboriginal and Torres Strait Islander people experience a burden of disease 2.3 times greater than that experienced by non‐Aboriginal Australians. Further, we know that factors contributing to the burden of disease have varied levels of impact, with the largest proportion attributed to cardiovascular disease (19%), followed by mental and substance use disorders (14%).^20^ To ensure adequate resources are available to address the burden caused by specific conditions, it may be expected that the proportion of research funding and effort in these areas would approximate the contributing burden proportion. While calls have been made for an increase in intervention publications,^17^ the complexities of factors affecting Aboriginal and Torres Strait Islander health require a wider scope of research designs to contribute to gaps in the knowledge base. It may be expected that a particular burden of disease will require descriptive research designs before intervention publications are possible. Geographic location has an impact on the burden of disease and remoteness contributes to the burden, with 1.4 times higher rates in remote and very remote areas compared with non‐remote areas.^21^ It could therefore be assumed that research outputs will vary in burden of disease focus and research designs in remote and non‐remote settings. This narrative review will provide an up‐to‐date overview of research output for Aboriginal and Torres Strait Islander health in Australia in line with the Closing the Gap reform priority of “being publicly accountable” to determine:

• the total number of peer‐reviewed publications related to Aboriginal and Torres Strait Islander health in Australia from 2008 to 2020; and
• the key characteristics of published Aboriginal and Torres Strait Islander health research and their relations, including:
  ▶ the research designs used;
  ▶ the jurisdiction and remoteness where the research was conducted; and
  ▶ the burden of disease or focus area of the research.

We aimed to identify all original peer‐reviewed health research conducted with Aboriginal and Torres Strait Islander peoples between 2008 and 2020. We conducted a systematic search of the literature via the Lowitja Institute website using the search tool Lowitja.search, which provides access to all Aboriginal and Torres Strait Islander health literature using the PubMed database. The listed topics in the database selected were “all” and “Aboriginal and Torres Strait Islander”; the search terms are summarised in the Supporting Information, section 1.

Publications were included if they presented original data on Aboriginal and Torres Strait Islander health in Australia and were published between January 2008 and December 2020, both inclusive. Reviews, editorials, commentaries, protocols, conference abstracts, and perspective articles were excluded. Articles relating to education or training of the health workforce were excluded if they did not directly relate to health outcomes. Those focused on parenting, violence and justice were also excluded unless linked to a specific burden of disease. Grey literature and government reports were also excluded.

Title and abstract screening were conducted independently by two authors (MB, JB) using Covidence (www.covidence.org). Disagreements for full text inclusion were discussed with a third author (SM). Given the large number of publications identified, the full text review and data extraction (Box 1) were independently conducted by one of three authors (MB, SM or KB) and then cross‐checked by another author (MB, SM, KB or RM). The quality checking of the data was conducted after full extraction by two authors (JB, BH).

Box 1 **Data coding and extraction details**

**Table:** 

**Data extracted**	**Details**
Article details	Year of publication
Design	Publications were categorised as analytical or descriptive using the Centre for Evidence Based Medicine study design tree.^22^ Analytical publications were further categorised as analytical observational, or experimental (randomised controlled trials or non‐randomised controlled trials)
Jurisdiction and geographical remoteness	For each publication, the states where data collection occurred were recorded (New South Wales [NSW], Victoria [VIC], Queensland,^23^ Tasmania [TAS], Australian Capital Territory [ACT], Western Australia [WA], South Australia [SA], Northern Territory [NT], or national). Publications conducted in the Torres Strait Islands were categorised as such. Publications that did not explicitly report the state where data collection was undertaken in (eg, implemented in five states) were coded as “not stated”
Burden of disease focus, or area of research focus	Each article was screened for primary outcome to identify the burden of disease focus according to the Australian Institute of Health and Welfare definitions of burden of disease categories.^24^ These categories quantify the fatal and non‐fatal effects and causes of diseases and injuries in the Australian population. Articles that could not be categorised into a burden of disease category were coded into author‐derived areas of research focus by three authors (MB, SM, KB). Categories were deductively coded into the following nine categories (Supporting Information, table 1): • health service planning delivery and improvement; • health and wellbeing; • nutrition; • mortality and morbidity; • racism; • medication; • physical activity; • genomics; and • multiple chronic diseases (which included publications that addressed multiple chronic diseases and their implications on health)
	It is acknowledged that some publications may fit into multiple categories — publications were categorised based on the most appropriate fit based on primary outcome measures

The search results are outlined in Box 2, using the Preferred Reporting Items for Systematic Reviews and Meta‐Analysis four‐phase flow diagram. A total of 6939 records were initially retrieved from the database searches. Following the removal of duplicate records, 6912 titles and abstracts were screened for eligibility, with 3630 records included in the full text review. Overall, 2150 articles were identified and included in the review (Box 2).

Box 3 presents the number of publications over time. Publications increased steadily in number, from 85 in 2008 to a high of 269 in 2019, before declining to 151 in 2020.

Box 2 **Preferred reporting items for systematic reviews and meta‐analysis four‐phase flow diagram**

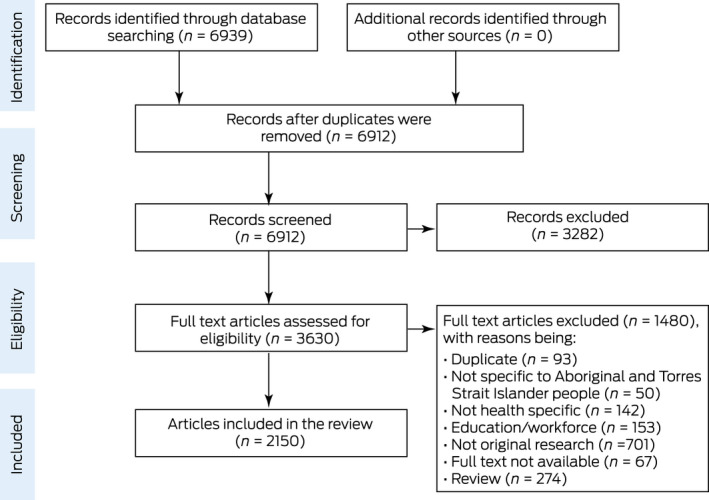

Box 3 **Number of Aboriginal and Torres Strait Islander health research articles meeting review inclusion criteria from 2008 to 2020 (*n* = 2150)**

**Figure:**
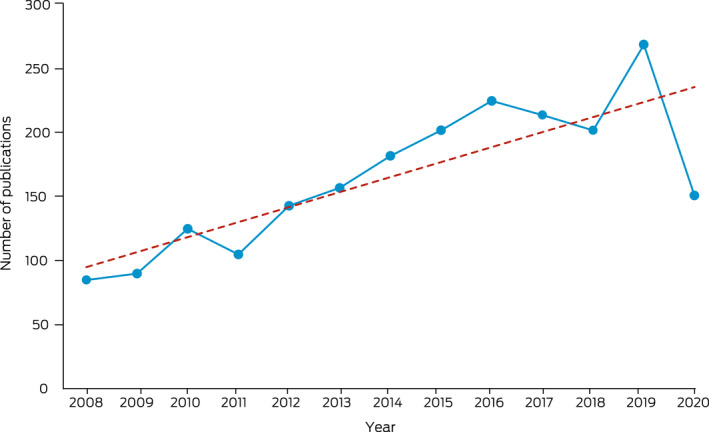
The dotted line in the graph represents the trend line.

## Key characteristics of published Aboriginal and Torres Strait Islander research

Box 4 presents the study designs used in the publications according to the Centre for Evidence‐Based Medicine study design hierarchy.^22^ Most publications used descriptive designs (*n* = 1250, 58.1%) and a smaller proportion used analytical observational designs (*n* = 841, 39.1%) and analytical experimental designs (*n* = 59, 2.7%). Of the analytical experimental publications, there were 57 randomised controlled trials (Box 4).

Box 4 **Number of publications using descriptive, analytical observational and analytical experimental designs by year (*n* = 2150)**

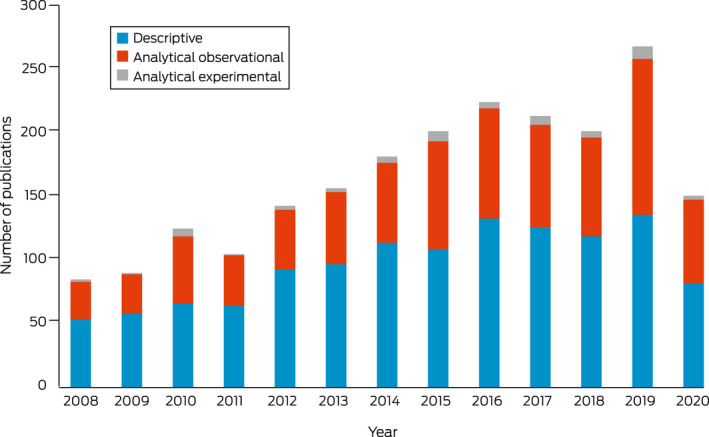

### Jurisdiction and geographical remoteness

Box 5 presents data on the jurisdiction and geographical remoteness of publications. The highest number of publications were conducted in the Northern Territory (*n* = 669, 25.9%), followed by Queensland (*n* = 449, 17.4%), Western Australia (*n* = 393, 15.2%) and New South Wales (*n* = 391, 15.1%). Only 9.3% of the publications were conducted nationally across all jurisdictions (*n* = 241). The highest number of publications were conducted in remote areas (*n* = 771, 28.8%) in the NT. Evidence of under‐reporting of remoteness was identified, with 22.2% of publications failing to articulate these details (Box 2).

Box 5 **Number of publications by jurisdiction and remoteness**

**Table:** 

**Category**	***n* (%)**
Jurisdiction*	
Northern Territory	669 (25.9%)
Queensland	449 (17.4%)
Western Australia	393 (15.2%)
New South Wales	391 (15.1%)
National	241 (9.3%)
South Australia	197 (7.6%)
Victoria	99 (3.8%)
Australian Capital Territory	23 (0.9%)
Torres Strait Islands	19 (0.7%)
Tasmania	14 (0.5%)
Not stated	88 (3.4%)
Remoteness^†^	
Remote	771 (28.8%)
Urban	499 (18.6%)
Regional	257 (9.6%)
Rural	254 (9.5%)
National	102 (3.8%)
Not stated	596 (22.2%)
Other	201 (7.5%)

* Studies were coded across multiple categories if they were conducted in more than one state, but not nationally. Therefore, numbers do not add to 2150.

† Studies were coded across multiple categories if they were conducted in different areas of remoteness, but not nationally. Numbers therefore do not add to 2150.

### Burden of disease

Box 6 presents the number of publications by burden of disease categories and research design. Overall, 1574 articles (73.2%) were able to be coded into a burden of disease category. The largest number of publications focused on mental and substance use disorders (*n* = 322; 20.5%), of which 77% (*n* = 247) were descriptive and only eight analytical experimental publications were conducted. Furthermore, infectious diseases (*n* = 222, 14.1%) had 50% (*n* = 111) of the publications using descriptive designs and 46% (*n* = 103) were analytical observational. Analytical observational designs were used more frequently than descriptive designs when addressing particular burdens of disease, such as kidney and urinary (78%), respiratory (69%), cancer (65%) and endocrine (61%). Three burdens of disease had a balanced design output; these included hearing and vision (54%), blood and metabolic (50%), and infant and congenital (50%). Less than 1% of the research examined skin or gastrointestinal disorders. The highest reported experimental publications were in oral disorders (*n* = 15, 16%) (Box 6).

Box 6 **Number of publications by burden of disease category and research design, 2008–2020 (*n* = 1574)**

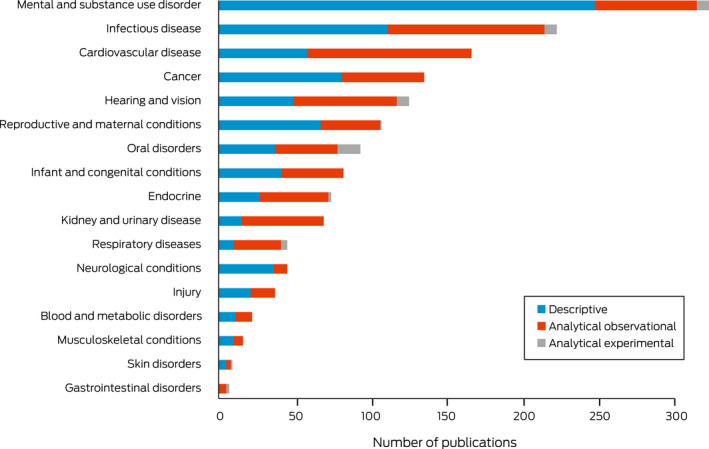

While the geographical remoteness is frequently not reported, studies in remote settings dominate across all areas of research and burden of disease categories (Box 7). Studies have been conducted across remote, rural, regional and urban settings for all burdens of disease, apart from skin and gastrointestinal disorders. National publications were limited across all burdens of disease, with the largest number of national publications reporting on mental and substance use disorders (*n* = 21, 5%). Research in a remote setting had higher proportions for infectious diseases (*n* = 104, 40%), hearing and vision (*n* = 72, 45%), and endocrine (*n* = 34, 40%) and respiratory diseases (*n* = 20, 39%).

Box 7 **Burden of disease by remoteness**

**Table:** 

**Burden of disease category**	**Remote**	**Rural**	**Regional**	**Urban**	**National**	**Other**	**Not stated**
Mental and substance use disorders	92 (5.8%)	44 (2.8%)	42 (2.7%)	75 (4.8%)	21 (1.3%)	27 (1.7%)	105 (6.7%)
Infectious diseases	104 (6.6%)	18 (1.1%)	18 (1.1%)	25 (1.6%)	9 (0.6%)	20 (1.3%)	63 (4.0%)
Cardiovascular disease	51 (3.2%)	22 (1.4%)	12 (0.8%)	47 (3.0%)	6 (0.4%)	11 (0.7%)	57 (3.6%)
Cancer	23 (1.5%)	22 (1.4%)	24 (1.5%)	34 (2.2%)	5 (0.3%)	19 (1.2%)	52 (3.3%)
Hearing and vision	72 (4.6%)	10 (0.6%)	14 (0.9%)	25 (1.6%)	7 (0.4%)	9 (0.6%)	22 (1.4%)
Reproductive and maternal conditions	44 (2.8%)	9 (0.6%)	12 (0.8%)	36 (2.3%)	1 (0.1%)	6 (0.4%)	24 (1.5%)
Oral disorders	24 (1.5%)	17 (1.1%)	10 (0.6%)	17 (1.1%)	6 (0.4%)	23 (1.5%)	15 (1.0%)
Infant and congenital conditions	27 (1.7%)	5 (0.3%)	8 (0.5%)	26 (1.7%)	3 (0.2%)	9 (0.6%)	17 (1.1%)
Endocrine diseases	34 (2.2%)	7 (0.4%)	6 (0.4%)	11 (0.7%)	2 (0.1%)	8 (0.5%)	17 (1.1%)
Kidney and urinary diseases	31 (2.0%)	16 (1.0%)	7 (0.4%)	18 (1.1%)	5 (0.3%)	4 (0.3%)	18 (1.1%)
Neurological conditions	17 (1.1%)	7 (0.4%)	11 (0.7%)	14 (0.9%)	1 (0.1%)	1 (0.1%)	9 (0.6%)
Respiratory diseases	20 (1.3%)	3 (0.2%)	2 (0.1%)	6 (0.4%)	0 (0.0%)	6 (0.4%)	14 (0.9%)
Injury	14 (0.9%)	7 (0.4%)	9 (0.6%)	9 (0.6%)	2 (0.1%)	4 (0.3%)	12 (0.8%)
Blood and metabolic disorders	6 (0.4%)	1 (0.1%)	3 (0.2%)	1 (0.1%)	0 (0.0%)	1 (0.1%)	10 (0.6%)
Musculoskeletal conditions	5 (0.3%)	4 (0.3%)	3 (0.2%)	2 (0.1%)	2 (0.1%)	2 (0.1%)	2 (0.1%)
Skin disorders	7 (0.4%)	1 (0.1%)	0 (0.0%)	1 (0.1%)	0 (0.0%)	0 (0.0%)	1 (0.1%)
Gastrointestinal disorders	3 (0.2%)	2 (0.1%)	0 (0.0%)	0 (0.0%)	1 (0.1%)	1 (0.1%)	2 (0.1%)
Total*	574 (36.5%)	195 (12.4%)	181 (11.5%)	347 (22.0%)	71 (4.5%)	151 (9.6%)	440 (28.0%)

* Not all studies were able to be categorised into burden of disease. In addition, studies could have multiple levels of remoteness (ie, not mutually exclusive), which affects the numbers.

### Area of research focus

Box 8 and Box 9 present the 576 articles that could not be included in the burden of disease categories that were coded into areas of research focus. The largest of these publications related to health services planning, delivery and improvement (*n* = 193, 33.5%), followed by health and wellbeing (*n* = 170, 29.5%), nutrition (*n* = 85, 14.8%), mortality and morbidity (*n* = 31, 5.4%), racism (*n* = 26, 4.5%), multiple chronic diseases (*n* = 26, 4.5%), medication (*n* = 17, 3%), physical activity (*n* = 15, 2.6%), and genomics (*n* = 13, 2.3%). Notable increases in health services planning, delivery and improvement articles were seen in 2013, with the focus on health and wellbeing articles steadily increasing over time. While some research has reported racism in health since 2008, in 2019 there was a significant increase in publications with this focus. All research areas most frequently employed a descriptive research design, except those with a focus on mortality and morbidity which predominately used analytical observational designs (Box 8 and Box 9).

Box 8 **Number of publications by research area focus and research design, 2008–2020 (*n* = 576)**

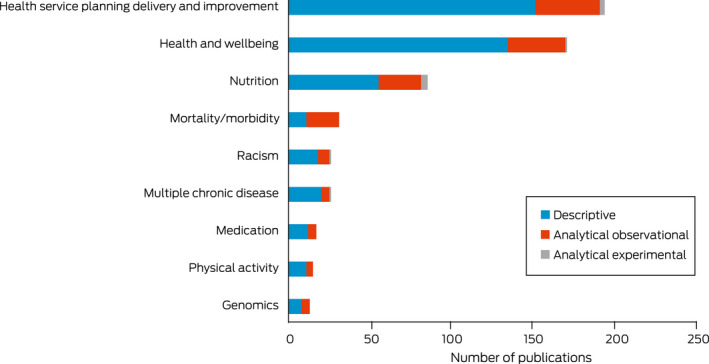

Box 9 **Area of research focus by year of publication, 2008–2020 (*n* = 576)**

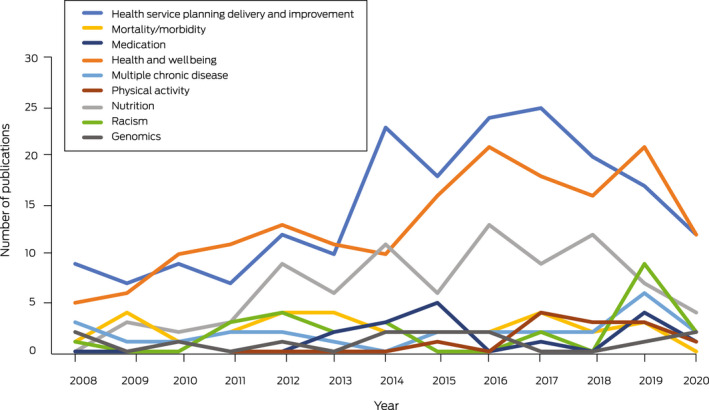

There is an under‐reporting of remoteness across areas of health focus. Studies in remote settings dominate most areas of research focus (Box 10). Research into racism is significantly under‐reported, with 37% not stated. Rural and regional settings are under‐represented except for health and wellbeing and health services planning, delivery and improvement. There are no national publications exploring medication, physical activity, or genomics.

Box 10 **Area of research focus by remoteness**

**Table:** 

**Research focus**	**Remote**	**Rural**	**Regional**	**Urban**	**National**	**Other**	**Not stated**
Health service planning delivery and improvement	73 (5.8%)	18 (3.1%)	30 (5.2%)	52 (9.0%)	14 (2.4%)	17 (3.0%)	49 (8.5%)
Health and wellbeing	53 (9.2%)	21 (3.6%)	25 (4.3%)	56 (9.7%)	9 (1.6%)	9 (1.6%)	47 (8.2%)
Nutrition	44 (7.6%)	9 (1.6%)	5 (0.9%)	15 (2.6%)	2 (0.3%)	3 (0.5%)	19 (3.3%)
Mortality/morbidity	6 (1.0%)	1 (0.2%)	3 (0.5%)	4 (0.7%)	1 (0.2%)	10 (1.7%)	11 (1.9%)
Multiple chronic diseases	7 (1.2%)	1 (0.2%)	3 (0.5%)	5 (0.9%)	2 (0.3%)	3 (0.5%)	6 (1.0%)
Racism	1 (0.2%)	1 (0.2%)	0 (0.0%)	7 (1.2%)	4 (0.7%)	4 (0.7%)	10 (1.7%)
Medication	3 (0.5%)	5 (0.9%)	4 (0.7%)	5 (0.9%)	0 (0.0%)	3 (0.5%)	6 (1.0%)
Physical activity	4 (0.7%)	3 (0.5%)	5 (0.9%)	6 (1.0%)	0 (0.0%)	0 (0.0%)	2 (0.3%)
Genomics	5 (0.9%)	0 (0.0%)	1 (0.2%)	2 (0.3%)	0 (0.0%)	1 (0.2%)	6 (1.0%)
Total*	196 (34.0%)	59 (10.2%)	76 (13.2%)	152 (26.4%)	32 (5.6%)	50 (8.7%)	156 (27.1%)

* The total number is greater than the number of research focus studies because some articles reported multiple levels of remoteness.

## Discussion

This narrative review aimed to describe research outputs specifically relating to Aboriginal and Torres Strait Islander people since the Closing the Gap targets were introduced by examining the number and key characteristics of publications. Original research outputs have continued to increase over time. There was a clear reduction in the number of publications in 2020, which may have been influenced by the impact of the coronavirus disease 2019 (COVID‐19) pandemic.^25^ Overall, more than 60% of the research conducted was descriptive, compared with 78% of research outputs identified in the 2006 review.^17^ However, this review added new details on research designs and found that particular diseases, such as kidney and urinary, respiratory, cancer and endocrine, are more likely to be examined using analytical observational designs, and that some burdens of disease have a balanced design output, including hearing and vision, blood and metabolic, and infant and congenital diseases. Descriptive research continues to be important in examining characteristics and determinants of health, with consideration of the diversity of Aboriginal and Torres Strait Islander peoples and their experiences of colonisation, dispossession and racism. Only 59 experimental publications have been conducted in the past 12 years, accounting for 2.7% of the research output. Conducting analytical research, particularly across multiple states and territories or nationally, is a complex and costly endeavour. Further, although Aboriginal and Torres Strait Islander health research ethics processes ensure culturally safe and appropriate research practice,^26^ conducting multisite ethics processes has been reported to affect timelines in trials.^27^ Despite experimental research being classified as the gold standard, recent reviews have identified knowledge and methodological gaps in documenting Aboriginal and Torres Strait Islander health research impact.^18^ Furthermore, leading Aboriginal and Torres Strait Islander academics are calling for a reimagining of health systems and research through a health justice framework^28^ to influence major changes to research priorities, including the research methods most highly valued.

Remote settings in the NT accounted for the largest proportion of geographic focus in this review. This finding aligns with previous reviews that have reported an under‐representation of research in urban settings.^14^, ^19^ However, the under‐reporting of research settings (20.2%) in the literature presents a challenge in interpreting these results. Future publications should ensure accurate reporting of the local context, including the research setting, to better determine geographical distribution of research focus. While the proportion of Aboriginal and Torres Strait Islander population living in remote settings (19%) is much higher than the proportion of non‐Aboriginal Australians living in remote settings (1.5%),^29^ the majority of Aboriginal and Torres Strait Islander people reside outside of remote locations. Therefore, while research is necessary to reduce health disparities present in remote settings, there is a clear need for increasing research relevant to urban, rural and regional settings to make a significant contribution to the overall health gap.

The National Health and Medical Research Council (NHMRC) Road Map 3 prioritises research to align with the highest burden of disease and those that are almost exclusive to Aboriginal and Torres Strait Islander people.^30^ This review found high research outputs in line with priority areas for health burdens relevant to remote settings, such as infectious diseases and hearing and vision. The Australian Institute of Health and Welfare reports that 94% of vision loss in Aboriginal and Torres Strait Islander people is preventable or treatable.^31^ Moreover, infectious diseases are 1.9 times greater in remote settings, and previous pandemics, such as the 2009 H1N1 influenza pandemic, had disproportionate impacts in remote settings.^32^ We identified research outputs related to all Australian Institute of Health and Welfare Burden of Disease categories.^33^ The largest proportion was attributed to mental and substance use disorders, including conditions such as anxiety and depressive disorders, drug and alcohol misuse, and tobacco use. The focus of this health burden correlates with the highest burden being cardiovascular disease and mental and substance use disorders, but given the high proportion of descriptive research (77%), there may be limitations to the impact of these outputs translating to improved health outcomes. As stated in an article published in 2020, “This story of failure and failing health has been told countless times in health and medical journal publications, and despite growing more frequent in number, these contributions to new knowledge never seem to translate to improved health outcomes”.^34^

Notably, we found that 27% of research conducted is not directly related to a specific burden of disease. Over time, there has been a growth in research more aligned with the holistic definition of Aboriginal health. Improvements to health services and health and wellbeing were the focus of research, accounting for 63% of research examining the broader consideration for health rather than a specific burden of disease. In 2019, there was a significant increase in research focused on racism in health, although racism has been reported in Aboriginal and Torres Strait Islander health since Closing the Gap was established. In 2020, the Black Lives Matter movement received global attention. A 2021 article reported, “Racism is a public health emergency of global concern”.^35^ However, this global attention was directed to “disparities in death and disease between black and white people in the USA” and seldom focused on indigenous peoples globally.^35^ More than 20 United Nations leaders signed a joint personal letter urging the UN to act against systemic racism.^36^ However, in Australia, Aboriginal and Torres Strait Islander people have been calling out systemic racism since colonisation, which can be traced through all Royal Commissions into Aboriginal and Torres Strait Islander peoples. At present, the impact of racism on the health and wellbeing of Aboriginal and Torres Strait Islander people cannot continue to be silenced^37^ and must be addressed in all research aiming to improve health.

With new investments in Aboriginal and Torres Strait Islander health being implemented and the updated NHMRC Road Map 3 highlighting the importance of Aboriginal and Torres Strait Islander researchers, community leadership and research priority including social and cultural determinants of health,^30^ we anticipate future outputs for Aboriginal and Torres Strait Islander health research to change significantly from this review. As published in 2020, “What is required is a broadening of our intellectual investment in Indigenous health: one that invites social scientific perspectives about the social world that Indigenous people occupy and its role in the production of illness and inequalities”.^28^ We need multidisciplinary teams led by Aboriginal and Torres Strait Islander people and driven by community priorities that demand transformational change in health and health systems. Research in Aboriginal and Torres Strait Islander health must move beyond deficit‐descriptive statistical portraits and rather interrogate and hold to account health systems. We acknowledge that publication frequency is not a catalyst for change alone and join calls for intellectual investment that prioritises Aboriginal and Torres Strait Islander ways of knowing, being and doing, and acknowledges the historical and contemporary colonisation, dispossession and racism that continue to have an impact on health outcomes today.

## Open access

Open access publishing facilitated by The University of Newcastle, as part of the Wiley ‐ The University of Newcastle agreement via the Council of Australian University Librarians.

## Competing interests

No relevant disclosures.

## Provenance

Not commissioned; externally peer reviewed.

## Supporting information

Supplementary results
