# Supplementary material for: Interrogating the intentions for Aboriginal and Torres Strait Islander health: a narrative review of research outputs since the introduction of Closing the Gap
Source: Med J Aust. 2022 Jun 10;217(1):50–7. doi: 10.5694/mja2.51601 (PMC9545599; doi:10.5694/mja2.51601)
Supplement: Supplementary file 1 — Supplementary results [file MJA2-217-50-s001.pdf]

Supporting Information

Supplementary material

This appendix was part of the submitted manuscript and has been peer reviewed. It is posted as supplied by the authors.

Appendix to: Kennedy M, Bennett J, Maidment S, et al. Interrogating the intentions for Aboriginal and Torres Strait Islander health: a narrative review of research outputs since the introduction of Closing the Gap. *Med J Aust* 2022; doi: 10.5694/mja2.51601.

Section 1

The Lowitja predefined terms for "Aboriginal and Torres Strait Islander Health" were used:

(((((australia[mh] OR australia*[tiab]) AND (oceanic ancestry group[mh] OR aborigin*[tiab] OR indigenous[tw])) OR (torres strait* islander*[tiab])) AND medline[sb]) OR ((((au[ad] OR australia*[ad] OR australia*[tiab] OR northern territory[tiab] OR northern territory[ad] OR tasmania[tiab] OR tasmania[ad] OR new south wales[tiab] OR new south wales[ad] OR victoria[tiab] OR victoria[ad] OR queensland[tiab] OR queensland[ad]) AND (aborigin*[tiab] OR indigenous[tiab])) OR (torres strait* islander*[tiab])) NOT medline[sb]) AND English[la])

Table 1

Category	Justification	Description
Health and Wellbeing	*More holistic factors of health – including social and environmental health that are otherwise not categorizable against burden of disease but acknowledged by Aboriginal and Torres Strait Islander people as central to definition of health.*	• Housing,<br>• Health promotion + programs *(publications outlining implementation/efficacy of programs, health promotion/ health education programs, telehealth, mobile ap service),*<br>• Empowerment & Resilience<br>• Identification *(Aboriginal and Torres Strait Islander checks, identification as barrier to health etc)*<br>• Health and wellbeing *(as a general theme)*<br>• Women's and Men's health *(identified specifically in papers)*<br>• Social & Emotional Wellbeing<br>• Connection and care for country<br>• Elder influence *(identified specifically in papers)*

		• Community connectedness + impact health
Health service planning delivery and improvement	*Important focus of Indigenous health is the adequacy of our health services and its ability to meet the cultural, biomedical and holistic*<br>*needs of Indigenous health.*	• Continuous quality improvement,<br>• Evaluations of health service use and health service systems,<br>• Cost analysis of programs/ health services,<br>• Cost of hospitalisation,<br>• Health service planning,<br>• Health care programs (Palliative care, Cancer specific care programs, aged care, coordination of care across services),<br>• Emergency department presentations,<br>• Health screening,<br>• Electronic data collection suitability,<br>• Socioeconomic disparity,<br>• Cost-consequence analysis of health care services,<br>• Health care expenditure,<br>• Culturally appropriate care/services,<br>• Economic evaluations,
Medication	*Didn't fit with other health burdens but directly linked to*	• Traditional medicines,<br>• Medication adherence/uptake,

	improving access to health care and treatment	• Medication cost and availability,<br>• Home medicines,<br>• Perspectives on medication,<br>• Medication in incarceration,
Racism	*Racism is a contributing factor on Aboriginal and Torres Strait Islander health.*	• Race and racism and its effect on health,<br>• Validation of racism measurement screening tools,<br>• Incidence,<br>• Experience in health,<br>• Health care discrimination and impacts in health etc.
Mortality / Morbidity	*Could not be categorised to specific burden of disease, but mortality and morbidity is measured and monitored in Closing the Gap.*	• Mortality and morbidity incidences and patterns, life expectancy, premature death.
Nutrition	*Category didn't fit with other burdens of health but relevant to consider given the burden of obesity.*	• Food supply,<br>• Intake and diet analysis,<br>• Dietary balance,<br>• Diet evaluations,<br>• Vitamin and nutritional deficiencies,<br>• Community gardens and capacity building of nutrition,<br>• Safe food practices,
Physical activity	*Did not address a specific health burden but themes were based on*	• Rates, adherence,<br>• Effect on wider health,

	health outcomes.	• Sedentary time compared to active time,<br>• Barriers and facilitators to sport involvement,<br>• Community perceptions of sport,
Genomics	*Did not have a specific health burden addressed, but was health focused.*	• Study of genes,<br>• Genomic sequencing,<br>• Biobanking,<br>• Genetic counselling for Indigenous people (cultural sensitivity within this area),<br>• Prevalence/specific genomes in Indigenous nation groups.
Multiple chronic diseases	*Publications that didn't have one specific health burden category but addressed "chronic disease" as a health issue.*	• Chronic disease often included many variations of diseases (cancer, diabetes, renal disease etc – without one singular disease focus)

Burden of disease categories:

Blood and Metabolic Disorders

Cancer

Cardiovascular Diseases

Endocrine Disorders

Gastrointestinal Disorders

Hearing and Vision Disorders

Infant and Congenital Conditions

Infectious Disease

Injury

Kidney and Urinary Diseases

Mental and Substance Use Disorders

Musculoskeletal Conditions

Neurological Conditions

Oral Disorders

Reproductive and Maternal Conditions

Respiratory Diseases

Skin Disorders
